# The prevalence and associated factors of third-trimester pregnancy depression in pre-pregnancy overweight and obesity women: a cross-sectional study in Guangdong, China

**DOI:** 10.3389/fpubh.2025.1687185

**Published:** 2025-10-14

**Authors:** Zheng Yao, Yanli Zhou, Jing Chen, Xuantian Liu, Danying Li, Jinguo Zhai

**Affiliations:** ^1^School of Nursing, Southern Medical University, Guangzhou, China; ^2^Nanfang Hospital, Southern Medical University, Guangzhou, China; ^3^Shenzhen Hospital of Southern Medical University, Shenzhen, China

**Keywords:** overweight, obesity, depression, the third trimester, lifestyle

## Abstract

**Background:**

The third trimester of pregnancy is the most frequent period of prenatal depression. Its prevalence is associated with differences in weight status. Pre-pregnancy overweight and obesity (OWOB) pregnant women have a high risk of third-trimester pregnancy depression. The aim of this study was to investigate the depression status of pre-pregnancy OWOB women in the third trimester of pregnancy, and to explore the influence of modifiable lifestyle behaviors during pregnancy on their depression status, so as to provide scientific basis for the prevention and intervention of third-trimester pregnancy depression in OWOB pregnant women.

**Methods:**

A cross-sectional survey was conducted among 441 pregnant women with pre-pregnancy OWOB recruited from two tertiary hospitals in Guangdong Province. Participants completed a basic information form, the Self-Rating Depression Scale, the Food Frequency Questionnaire, the Perinatal Pregnant Women’s Health Literacy Scale, the Physical Activity Rating Scale, and the Pittsburgh Sleep Quality Index. Binary logistic step regression was used to identify the associated factors of pregnancy depression in the third trimester.

**Results:**

Among the 441 participants, 411 (93.20%) were overweight and 30 (6.80%) were obesity prior to pregnancy. The mean score of depression was 48.50 ± 9.48. A total of 289 (65.53%) women had no depression, while 107 (24.26%) had mild, 44 (9.98%) had moderate, and 1 (0.23%) had severe depression. Binary logistic regression analysis showed that educational level, career, family income, and first pregnancy or not were the influencing factors for third-trimester pregnancy depression among pre-pregnancy OWOB women. The balanced dietary pattern, higher physical activity level and health literacy were protective factors, while the highly processed dietary pattern and sleep disorders were risk factors (*p* < 0.05).

**Conclusion:**

About one-third of pre-pregnancy OWOB pregnant women experienced depression in the third trimester of pregnancy. The depression was most likely associated with modifiable lifestyle behaviors. Although the cross-sectional design may not necessarily indicate a cause-effect relationship, the findings suggest that early lifestyle interventions may still be an effective strategy for improving mental health outcomes in this high-risk population.

## 1 Introduction

Overweight and obesity (OWOB) have become increasingly serious public health concerns worldwide. According to the World Health Organization (WHO), the global prevalence of obesity rose from 4.7% in 1975 to 16% in 2022, with 44% of adult women overweight (1). China has the highest number of individuals with obesity in the world (2). By 2030, China’s medical expenses caused by OWOB will reach 417.8 billion CNY, accounting for 21.5% of total national healthcare expenditures (3–6), and the economic burden will be huge. This trend is also evident among women of childbearing age. A national study conducted across 31 provinces in mainland China reported 24.5% overweight rate and 9.0% obesity rate among women aged 18–44 years (7), with 25.1% pregnant women overweight (8). In this regard, the National Health Commission, together with 15 other ministries, launched the “Weight Management Year” campaign (9). Additionally, the Chinese Nutrition Society published the Dietary Guidelines for Pregnant and Lactating Women (2022), with the core of achieving a normal pre-pregnancy weight (10). These actions reflect growing national attention to weight management in pregnant women.

In 2024, the National Health Commission and other three departments jointly issued the Guidelines on Promoting the Development of Fertility-Friendly Hospitals, which explicitly proposed to include prenatal depression screening into routine maternal healthcare services (11). Maternal mental health issues are also included in national health policy priority areas. The third trimester of pregnancy, defined as the period from 28 weeks of gestation to delivery, is considered the peak period for prenatal depression (12). In China, the prevalence of depression among the third-trimester pregnant women ranged from 12.01 to 44.4% (13, 14), posing significant risks to maternal well-being, neonatal outcomes and even family functioning (15, 16). Previous studies have shown that OWOB pregnant women are more susceptible to negative emotion such as depression and anxiety (17). On the one hand, excessive weight is associated with metabolic abnormalities and increased risk of pregnancy complications; on the other hand, these women may experience heightened body image concerns, social stigma, and health-related anxiety. The combined burden of physical and psychological stressors contributes to a higher risk of depression (18). Given the rising proportion of OWOB women among the pregnant population, investigating the prevalence and influencing factors of third-trimester depression in this group is of great importance, so as to inform the early identification of high-risk individuals and support the development of targeted personalized interventions.

Previous studies also identified that lower education level and family income, advanced maternal age, unemployment and other demographic factors are predictors of depression in third trimester of pregnancy (19–21). Gravidity, gestational diabetes mellitus, gestational hypertension, and other pregnancy complications and other obstetric variables are also closely related to the occurrence of depression (22). However, most existing studies have not stratified participants according to the body mass index (BMI), and pregnant women with underweight, normal weight, overweight, and obesity are often analyzed as a single group, without accounting for the potential differential impact of BMI categories on mental health. Considering that OWOB may elevate the risk of prenatal depression through multiple pathways (including metabolic abnormalities, chronic inflammation, psychological stress, etc.), such undifferentiated approaches may obscure the specific mechanism and risk profiles unique to OWOB pregnant women. Therefore, it is necessary to investigate third-trimester depression specifically in pre-pregnancy OWOB women, and further clarify the high-risk features and intervention priorities of this population.

Meanwhile, as the research on “lifestyle medicine” continues to deepen, researchers have increasingly recognized the important role of lifestyle in mental health and the prevention and treatment of depression (23–25). In terms of diet, Silva’s review of 10 studies found a negative association between adherence to a “healthy dietary pattern” and perinatal depression (26). Regarding sleep, Poeira noted that poor sleep quality during pregnancy predicts prenatal depression, with sleep quality progressively deteriorating as gestational age increases (27). Reduced physical activity during pregnancy has also elevates depression risk (28). Furthermore, a study of 1,478 pregnant women in Beijing found that passive smoking increased the risk of prenatal depression by 1.85 times, with husband-related exposure playing an important role (29). Health literacy refers to the ability of individuals to obtain, understand and apply health information to maintain and promote health. It is the basis of health-related behaviors and affects lifestyle choices. Studies have shown that lacking health literacy can negatively predict the occurrence of depression during pregnancy (30). These unhealthy lifestyle behaviors may contribute to depression through common biological pathways, including disrupting the hypothalamic–pituitary–adrenal (HPA) axis, increasing cortisol, low-grade systemic inflammation, and oxidative stress, and resulting in neurodegeneration and reduced neurogenesis (31, 32). OWOB, as inflammatory disease of adipose tissue, further enhance the production of pro-inflammatory cytokines, which in turn affect neurotransmitter expression and promote the development of depression (33). Notably, pre-pregnancy OWOB women often exhibit one or more unhealthy lifestyle behaviors, such as excessive intake of high-calorie and high-fat foods, physical inactivity, etc., which substantially heightens their risk of prenatal depression. Thus, examining the relationship between lifestyle behaviors and depression during third-trimester pregnancy among this specific population may help identify feasible avenues to improve their physical health and thus alleviate adverse psychological outcomes.

Therefore, the aim of this study was to assess the prevalence and influencing factors of depression during the third trimester of pregnancy among pre-pregnancy OWOB women, in order to provide evidence for the development and implementation of targeted prevention and intervention strategies for depression in pre-pregnancy OWOB pregnant women.

## 2 Methods

### 2.1 Study design

The study adopted a cross-sectional design and a convenience sampling method. Data were collected from February 2023 to September 2024. Based on geographical location and convenience of implementation, Guangzhou city and Shenzhen city were selected as representatives of Guangdong Province. Participants were recruited from the obstetric outpatient departments of Nanfang hospital Southern Medical University and Shenzhen hospital of Southern Medical University, both of which are the tertiary general teaching hospitals. Two trained researchers screened the electronic medical records of pregnant women attending routine prenatal examinations in the waiting areas. Eligible individuals were consecutively invited to participate until the required sample size was reached. Two trained researchers explained the study’s objective, content, and cooperation to the participants. Then participants signed the informed consent and completed several electronic questionnaires. This study has been registered in the clinical trial registry, with the registration number: ChiCTR2300077450.

### 2.2 Study population

Calculate the sample size required for the study. As no prior studies have reported the prevalence of third-trimester depression specifically among pre-pregnancy OWOB pregnant women in China, given the fact that pre-pregnancy OWOB pregnant women may face a higher risk of depression in the third trimester of pregnancy than general pregnant women, we referred to the findings of Shen (34), which indicated that the prevalence of depression in high-risk pregnant women was 29.3%. According to the cross-sectional survey sample size calculation formula (35), set the two-sided significance level (*α*) of 0.05 and a margin of error (*δ*) of 0.05, the required sample size was: *n* = 1.96^2^ × 0.293 × (1–0.293) / 0.05^2^ ≈ 319. Considering a potential 20% dropout or error rate, 319/(1–0.20) ≈ 399, the sample size required for this study is at least 399. A total of 441 participants were ultimately recruited.

Pre-pregnancy weight status was classified based on BMI according to WHO criteria. Following the recommended thresholds for the Chinese population proposed by the Chinese Obesity Working Group, pre-pregnancy overweight was defined as 24 kg/m^2^ ≤ BMI < 28 kg/m^2^, and pre-pregnancy obesity as BMI ≥ 28 kg/m^2^, and this overweight or obesity status would continue throughout pregnancy (36). Pre-pregnancy BMI was calculated as the self-reported pre-pregnancy weight divided by the square of the height measured at the first prenatal examination. For participants who were unsure of their pre-pregnancy weight or whose reported weight was deemed unreliable, BMI was calculated using the hospital-recorded weight measured at the first prenatal examination, provided that it was measured before 12 weeks of gestation (37). Participants need to wear light clothes and no shoes when weighing.

The target population was determined to be pregnant women over 18 years old with pre-pregnancy BMI ≥ 24 kg/m^2^, diagnosed as singleton intrauterine pregnancy, no signs of labor or abortion, and had adequate reading and communication abilities. According to the definition of the third trimester of pregnancy, only women with gestational age of ≥ 28 weeks were considered. However, since this study used the Pittsburgh Sleep Quality Index and the Physical Activity Rating Scale to assess sleep quality and physical activity over the past month (i.e., 4 weeks), we restricted the inclusion to women between 32 weeks of gestation and before delivery to ensure that these behaviors reflected the third-trimester pregnancy. Women with a history of severe hypertension, heart disease, diabetes, pre-pregnancy depression or other diagnosed mental illness were excluded.

### 2.3 Data collection

In the Basic information form, participants provided detailed demographic information such as age, ethnicity, education level, career, marital status, monthly per capita family income (CNY), and residence. Medical status was collected through medical records, including whether it was the first pregnancy, and whether there was gestational diabetes mellitus or gestational hypertension.

The Self-Rating Depression Scale (SDS) by Zung was used to assess depression over the past week (38). It has been demonstrated as a valuable and effective tool for detecting depression in pregnancy (39). The scale includes four dimensions with 20 items. A 4-point scale was used for each entry. The higher the score, the more severe the depression tendency. A total score ≤ 52 is no depression, 53 ~ 62 is mild depression; 63 ~ 72 is moderate depression; and ≥ 73 is severe depression. The Cronbach’ s *α* coefficient of this scale was 0.86 (40). The Cronbach’s α coefficient for the scale in this study was 0.74.

The Food Frequency Questionnaire (FFQ) represents a shortened version of the validated semi-quantitative FFQ for Chinese pregnant women and was revised to incorporate the dietary habits of the Guangdong region (41, 42). The questionnaire consisted of 23 food categories. According to previous researches, for most foods, the frequency division is more applicable with a finer division scale at high frequency levels, and if the intake frequency of a food is less than once a month, the contribution of this food to nutrient intake will also be negligible (43), and to reduce recall bias, pregnant women’s dietary habits over the past week were investigated. Therefore, we determined the frequency as: ① no eating/drinking; ② 1 ~ 2 times a week; ③ 3 ~ 4 times a week; ④ 5 ~ 6 times a week; ⑤ once a day; and ⑥ ≥ 2 times a day. Food intake: not eaten in the last week, ≤ 50 g, 100 g, 150 g, 200 g, and ≥ 250 g. Daily intake of each food (g/d) = average daily intake × total weekly intake frequency / 7. Each individual has a factor score on each dietary pattern (DP). The higher the score, the more inclined to this DP. The Cronbach ‘s *α* coefficient of the questionnaire in this study was 0.86, the content validity was 0.80.

The Perinatal Pregnant Women’s Health Literacy Scale was compiled by Wang (44) in 2017, including three dimensions of functional health literacy, communicative health literacy, and critical health literacy, 51 items. The scale uses the Likert 5-level scoring method, from “very disagree” to “very agree,” 1 to 5 points in turn. The higher the score, the higher the health literacy, and ≥ 204 points indicate having health literacy. The Cronbach’s *α* coefficient of the scale was 0.885, and the Cronbach’s α coefficients of each dimension were 0.802, 0.773 and 0.823. The Cronbach’s α coefficient of the scale in this study was 0.943.

The Physical Activity Rating Scale (PARS-3) was compiled by the Japanese scholar, Takao Hashimoto, and revised by Liang (45). The activity level was examined from 3 aspects: exercise intensity, exercise time and exercise frequency. Activity level = intensity × time × frequency. Each aspect was divided into 5 grades. Intensity and frequency were scored 1 ~ 5, and time was scored 0 ~ 4, so the highest amount of exercise is 100 points, the lowest is 0 points. Activity level was rated on a scale of low (≤ 19 points), moderate (20 ~ 42 points) and high (≥ 43 points). The retest reliability of the scale was 0.82, and Javalle’s research proved that the scale has high reliability (46). The Cronbach’s *α* coefficient for the scale in this study was 0.65.

The Pittsburgh sleep quality index (PSQI) is a subjective sleep quality assessment scale developed by Buysse (47) of the Pittsburgh Medical Center in 1989. It was translated into Chinese by Liu et al. in 1996 and applied in the Chinese population (48), with a Cronbach’s *α* coefficient of 0.84. The scale contains 7 dimensions, each dimension is scored from 0 to 3, and the cumulative score of each dimension is the total score of PSQI (0–21), and a higher score indicates poorer sleep quality. The Cronbach’s α coefficient of the PSQI was 0.83 in this study.

### 2.4 Data analysis

Epi Data 3.1 was used to establish database, with logic checks applied and double data entry conducted to ensure accuracy. SPSS 27.0 was used for data analysis. When analyzing DP, in order to reduce the number of variables and the variation degree of food intake among individuals, improving interpretability, according to the *Chinese Residents Balanced Diet Pagoda* and the relationships between food attributes and nutritional composition (49), 23 items were divided into 18 non-overlapping food groups (Supplementary Table S1). Factor analysis was used to analyze DP to reduce the dimensionality of food frequency questionnaire data and to identify underlying dietary patterns. KMO and Bartlett’s test of sphericity were used to determine whether the data was suitable for factor analysis. Main DPs were determined according to scree plot, the proportion of variance explained and the eigenvalue. According to nutritional knowledge and clinical plausibility of the retained factors number, this study selected the eigenvalue beyond the statistical cut-off value as the criteria to determine the number of retained DPs (*λ* > 1.4) (25, 50). To achieve a relatively simple and more interpretable factor structure, the maximum variance rotation of the initial factor load matrix is performed. Food groups with absolute factor loadings > 0.25 are generally considered to contribute meaningfully to a given factor (51); in this study, the retention standard of food group items in DP was set as 0.3 (52). DPs were then named after the combination of food groups with high factor loadings and relevant nutritional knowledge. After the DP was extracted, each participant had a corresponding DP score (factor score) for each pattern. The higher the score, the more consistent the individual’s dietary situation with the DP, and the score was then included in the logistic regression model.

Measurement data with a normal distribution were expressed in $\bar{X}\pm \mathrm{SD}$, while those with a non-normal distribution were expressed as *M* (*P*_25_, *P*_75_), and count data was expressed in rate (%). Independent samples t-test and one-way ANOVA were used to compare the data with normal distribution in different characteristic groups. Mann–Whitney U and Kruskal-Wallis H tests were used to compare the data with skewed distribution. Chi-square tests were used to compare categorical variables. Binary logistic regression analysis was performed to identify predictors of depression in third-trimester pregnancy, with the presence or absence of depression as the dependent variable. Independent variables included demographic and lifestyle-related factors that were statistically significant (*p* < 0.05) in univariate analysis. Odds ratios (ORs) and 95% confidence intervals (CIs) were calculated. *p* < 0.05 was considered statistically significant.

### 2.5 Rigour

Before the study commenced, all personnel involved in data collection received standardized training to ensure a consistent guidance explanation was used during the collection process. All variables were collected through self-administered online questionnaires completed by the participants. The questionnaire was developed using the online survey platform (Wenjuanxing platform), with restrictions to allow only one submission per device and account. All questions were set as mandatory to prevent missing data. Upon completion, the researchers checked each questionnaire for completeness and obvious errors. If any missing responses or inconsistencies were identified, participants were asked to provide corrections or complete the items immediately. After each questionnaire, there are participants’ contact information. When questionable data were identified, participants were contacted by phone for verification. After collection, two independent researchers checked and eliminated the questionnaires with the answer time < 15 min or more than 50% of the same responses in a row.

### 2.6 Ethics approval and consent to participate

All procedures in the study were conducted in accordance with the principles outlined in the Declaration of Helsinki. Ethical approval was obtained from both the Ethics Committee of Southern Medical University (Approval No. NFYKDX003-2023–64) and the Medical Ethics Committee of Nanfang Hospital, Southern Medical University (Approval No. NFEC-2024-493). Participants were informed of the study’s purpose, scope, ethical sensitivity, and potential benefits, assuring them that participation is voluntary. Before data analysis, participants were explicitly informed of their right to refuse participation or withdraw from the study. All participants provided written informed consent. According to the scale score, the participants who were judged for depression will be further provided with professional mental health resources by the hospital.

## 3 Results

A total of 461 questionnaires were distributed, and 441 questionnaires were left after deleting missing key variables or invalid questionnaires. The effective response rate was 95.66%. Finally, 441 pregnant women were included, with an average age of 31.15 ± 4.03 years. The average pre-pregnancy BMI was 25.44 ± 1.79 kg/m^2^. There were 411 (93.20%) women classified as overweight and 30 (6.80%) as obesity before pregnancy. Most participants had an undergraduate educational level or above (*n* = 169, 38.32%), and the majority were enterprises or institutions staff (*n* = 192, 43.75%). The mean SDS score of pre-pregnancy OWOB pregnant women was 48.50 ± 9.48. Of the participants, 289 (65.53%) had no depression, 107 (24.26%) had mild depression, 44 (9.98%) had moderate depression, and 1 (0.23%) had severe depression. Additional demographic characteristics of the participants are presented in Table 1. Univariate analysis showed that significant differences were observed between women with and without third-trimester depression in terms of educational level, career, family income, marital status, residence, and whether it was the first pregnancy (*p* < 0.05).

**Table 1:** Demographic data of pre-pregnancy OWOB pregnant women (*n* = 441).

Characteristics features	Total (%) (*n* = 441)	Depression (%) (*n* = 152)	Non-depression (%) (*n* = 289)	$\chi^{2}$	*P*
Maternal age (year)				2.272	0.166
< 35	354 (80.27)	128 (84.21)	226 (78.20)		
≥35	87 (19.73)	24 (15.79)	63 (21.80)		
Ethnicity				2.872^a^	0.055
Han ethnic	433 (98.18)	152 (100)	281 (97.23)		
Minorities	8 (1.82)	0 (0)	8 (2.77)		
Educational level				52.820	< 0.001
Junior high and below	67 (15.19)	44 (28.95)	23 (7.96)		
High school/secondary	74 (16.78)	37 (24.34)	37 (12.80)		
College	131 (29.71)	34 (22.37)	97 (33.56)		
Undergraduate and above	169 (38.32)	37 (24.34)	132 (45.68)		
Careers				35.598	< 0.001
Healthcare personnel	42 (9.38)	4 (2.63)	38 (13.15)		
Enterprises or institutions staff	192 (43.75)	51 (33.55)	141 (48.79)		
Self-employed	70 (15.63)	28 (18.42)	42 (14.53)		
Unemployed	87 (19.64)	48 (31.58)	39 (13.49)		
Other	50 (11.61)	21 (13.82)	29 (10.04)		
Monthly per capita family income				30.000	< 0.001
< 4,000 yuan	43 (9.75)	20 (13.16)	23 (7.96)		
4,000 ~ 5,999 yuan	122 (27.66)	61 (40.13)	61 (21.11)		
6,000 ~ 9,999 yuan	146 (33.11)	37 (24.34)	109 (37.72)		
10,000 ~ 14,999 yuan	69 (15.65)	24 (15.79)	45 (15.57)		
≥ 15,000 yuan	61 (13.83)	10 (6.58)	51 (17.65)		
Marital status				7.959	0.019
Unmarried	8 (1.81)	2 (1.31)	6 (2.08)		
Married	429 (97.28)	146 (96.05)	283 (97.92)		
Divorced	4 (0.91)	4(2.64)	0 (0)		
Residence				8.446	0.015
City	252 (57.14)	79 (51.97)	173 (59.86)		
Town	69 (15.65)	19 (12.50)	50 (17.30)		
Rural	120 (27.21)	54 (35.53)	66 (22.84)		
First pregnancy or not				6.036	0.014
Yes	186 (42.18)	52 (34.21)	134 (46.37)		
No	255 (57.82)	100 (65.79)	155 (53.63)		
Gestational hypertension				0.298	0.585
Yes	27 (6.37)	8 (5.26)	19 (6.57)		
No	414 (93.63)	144 (94.74)	270 (93.43)		
Gestational diabetes mellitus				0.824	0.364
Yes	104 (23.77)	32 (21.05)	72 (24.91)		
No	337 (76.23)	120 (78.95)	217 (75.09)		

Chi-square test was performed, and the χ^2^ value was reported.

**Figure 1:**
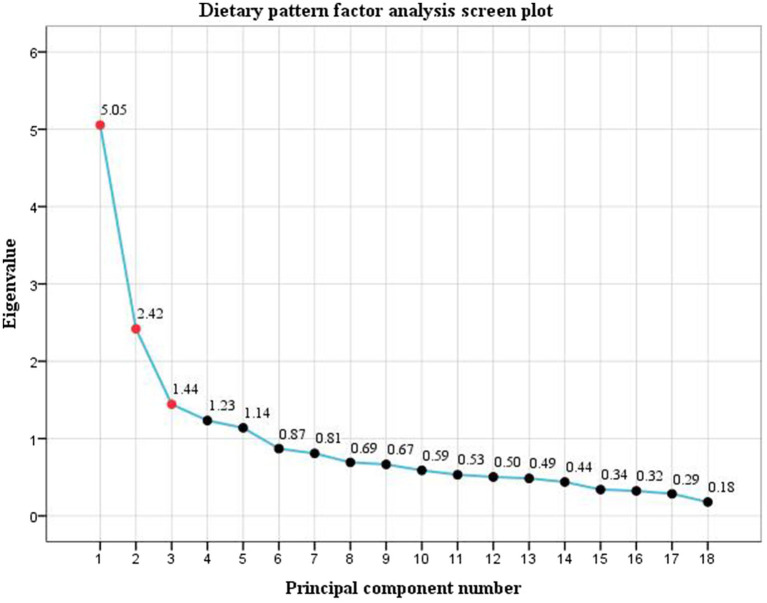
Principal components analysis gravel map of dietary pattern.

The factor analysis showed a KMO statistic of 0.793 and Bartlett’s test of sphericity *χ*^2^ = 2812.973 (*p* < 0.001). Three dietary patterns were extracted according to the eigenvalues, scree plot (Figure 1) and the proportion of variance explained. The cumulative variance contribution rate reached 49.54%, accounting for 18.14, 16.65, and 14.75%, respectively. The mean factor scores for each pattern among the participants were 0. The first DP is mainly composed of vegetables, beans, freshwater seafood, nuts, and grains and tubers, reflecting traditional eating habits in southern China, and was named the Traditional DP. The second DP included fruits, red meat, poultry, dairy products, eggs, rice/flour products, and seafood, representing a more nutritionally balanced composition suitable for maternal and fetal needs during pregnancy, which was named the Balanced DP. In the third pattern, the factor loads of fried and pickled foods, organ meats, and alcoholic and carbonated beverages were high, and most of them were highly processed foods, which were named highly processed DP. The food groups and factor loadings for each pattern are presented in Table 2. Spearman correlation analysis showed that pre-pregnancy BMI was negatively correlated with balanced DP (*r* = −0.263, *p* < 0.001), and positively correlated with traditional DP (*r* = 0.097, *p* = 0.043) and highly processed DP (*r* = 0.224, *p* < 0.001).

**Table 2:** Main food groups of three dietary patterns.

Item	Traditional DP		Balanced DP		Highly processed DP	
	Food	Factor loading	Food	Factor loading	Food	Factor loading
	Dark-colored vegetables	0.741	Fruits	0.766	Fried and pickled foods	0.807
	Light-colored vegetables	0.688	Red meat	0.682	Organ meats	0.723
	Bean products	0.574	Poultry	0.615	Alcoholic and carbonated beverages	0.690
	Grains and tubers	0.539	Dairy products	0.575	Edible mushrooms	0.583
	Nuts	0.535	Eggs	0.465	Processed snacks	0.531
	Freshwater seafood	0.531	Rice and flour-based food	0.425		
			Seafood	0.374		
Eigenvalues		5.054		2.419		1.444
Variance contribution rate		18.14%		16.65%		14.75%
Cumulative variance contribution rate		18.14%		34.79%		49.54%

DP, dietary pattern.

Among pre-pregnancy OWOB pregnant women, the mean PSQI score for sleep quality was 6.43 ± 2.97. The mean PARS-3 score for physical activity was 7.11 ± 5.47, and the mean score for perinatal health literacy was 197.04 ± 16.01. During the whole pregnancy, exposure to smoke from other smokers ≥ 1 day per week, with an average of ≥ 15 min each time, can be regarded as passive smoking (53). A total of 308 (69.84%) participants reported exposure to secondhand smoke during pregnancy, while 133 (30.16%) did not. Analysis showed that women with third-trimester pregnancy depression had significantly different scores in DPs, physical activity, sleep quality, and health literacy compared to those without depression, with all differences reaching statistical significance (*p* < 0.05). However, the presence of depression was not associated with passive smoking during pregnancy (Detailed results are presented in Table 3).

**Table 3:** Lifestyle status of pre-pregnancy OWOB pregnant women (*n* = 441).

Variable		Scores ($\bar{X}\pm \mathrm{SD}$, %)			$\chi^{2}$/*t*	*P*
		Total (*n* = 441)	Depression (*n* = 152)	Non-depression (*n* = 289)		
Traditional diet		0	−0.08 ± 1.02	0.04 ± 0.99	1.162^a^	0.043
Balanced diet		0	−0.33 ± 1.07	0.17 ± 0.92	4.952^a^	< 0.001
Highly processed diet		0	0.28 ± 1.38	−0.15 ± 0.68	−3.652 ^a^	< 0.001
Physical activity		7.11 ± 5.47	5.36 ± 2.92	8.03 ± 6.37	3.629 ^a^	< 0.001
Sleep quality		6.43 ± 2.97	7.08 ± 3.23	6.09 ± 2.76	−3.206 ^a^	0.002
Health literacy		197.04 ± 16.01	190.86 ± 13.73	200.30 ± 16.19	6.443 ^a^	< 0.001
Passive smoking^†^	Yes	308 (69.84)	104 (68.4)	204 (70.6)	0.222 ^b^	0.663
	No	133 (30.16)	48 (31.6)	85 (29.4)		

^†^During the whole pregnancy, exposure to smoke from other smokers ≥ 1 day per week, with an average of ≥ 15 min each time, can be regarded as passive smoking.^a^Indicates independent-samples *t* test (t value reported); ^b^Indicates chi-square test (*χ*^2^ value reported).

Binary logistic regression analysis was conducted using the third-trimester pregnancy depression as the dependent variable. Independent variables included demographic and lifestyle factors that were statistically significant (*p* < 0.05) in the univariate analysis. A forward stepwise selection method was applied. The results showed that among demographic factors, education level, career, monthly per capita family income, and whether it was the first pregnancy were influencing factors of third-trimester pregnancy depression in pre-pregnancy OWOB women. Among them, the risk of third-trimester depression for pre-pregnancy OWOB women with an educational level of junior high and below was 2.358 times that of undergraduate and above (95% *CI*: 1.368 ~ 5.744, *p* = 0.043), and the risk of depression for unemployed women was 7.622 times that of healthcare personnel women (95% *CI*: 1.703 ~ 34.110, *p* = 0.008). Regarding lifestyle behaviors, adherence to a balanced DP, higher levels of physical activity, and better health literacy were identified as protective factors, while adherence to a highly processed DP and poor sleep quality were identified as risk factors (*p* < 0.05). The binary logistic regression Hosmer and Lemeshow test showed *p* = 0.267 > 0.05 and the model was valid (Table 4).

**Table 4:** Influencing factors of third-trimester pregnancy depression in pre-pregnancy OWOB women.

Variable	*B*	SE	Wald	*P*	OR (95% CI)
Educational level			11.575	0.009^**^	
Undergraduate and above	Ref.				1.000
Junior high and below	0.858	0.454	3.563	0.043^*^	2.358 (1.368, 5.744)
High school/secondary	0.621	0.408	2.318	0.128	1.860 (0.837, 4.137)
College	−0.472	0.345	1.878	0.171	0.624 (0.317, 1.225)
Careers			12.069	0.017^*^	
Healthcare personnel	Ref.				1.000
Enterprises or institutions staff	1.711	0.681	6.307	0.012^*^	5.534 (1.456, 21.038)
Self-employed	1.069	0.748	2.040	0.153	2.913 (0.672, 12.630)
Unemployed	2.159	0.756	8.159	0.004^**^	8.659 (1.969, 38.088)
Other	2.031	0.765	7.057	0.008^**^	7.622 (1.703, 34.110)
Monthly per capita family income (yuan)			11.937	0.018^*^	
≥ 15,000	Ref.				1.000
< 4,000	0.047	0.647	0.005	0.942	1.048 (0.295, 3.727)
4,000 ~ 5,999	0.968	0.482	4.034	0.045^*^	2.633 (1.024, 6.773)
6,000 ~ 9,999	0.102	0.494	0.042	0.837	1.107 (0.420, 2.914)
10,000 ~ 14,999	0.945	0.484	3.815	0.051	2.573 (0.997, 6.642)
Marital status			1.444	0.486	
Unmarried	Ref.				1.000
Married	1.321	1.100	1.444	0.229	3.748 (0.434, 32.342)
Divorced	20.914	19954.788	< 0.001	> 0.999	-
Residence			1.219	0.544	
City	Ref.				1.000
Town	−0.277	0.389	0.506	0.477	0.758 (0.354, 1.625)
Rural	0.202	0.327	0.381	0.537	1.224 (0.644, 2.326)
First pregnancy or not					
Yes	Ref.				1.000
No	−0.732	0.281	6.799	0.009^**^	2.080 (1.199, 3.606)
Traditional diet	0.102	0.144	0.497	0.481	1.107 (0.835, 1.468)
Balanced diet	−0.611	0.143	18.278	< 0.001^***^	0.543 (0.410, 0.718)
Highly processed diet	0.518	0.143	13.136	< 0.001^***^	1.679 (1.269, 2.222)
Physical activity	−0.080	0.026	9.527	0.002^**^	0.923 (0.877, 0.971)
Sleep quality	0.128	0.046	7.894	0.005^**^	1.137 (1.039, 1.243)
Health literacy	−0.036	0.010	12.916	< 0.001^***^	0.965 (0.946, 0.984)
Constant	2.635	2.247	1.375	0.241	13.940

Dependent variable: 1 = depression; 0 = non-depression.

B, unstandardized regression coefficient; SE, standard error; OR, odds ratio; 95% CI, 95% confidence interval.

^*^P < 0.05; ^**^p < 0.01; ^***^p < 0.001.

## 4 Discussion

There is growing research in the field of prenatal mental health. However, pregnant women with pre-pregnancy OWOB as the vulnerable group to the mental disorder are still overlooked. This study investigated the association between demographic factors and depression in the third trimester of pregnancy among this high-risk group and comprehensively explored associations with various modifiable lifestyle factors, including dietary patterns, sleep quality, physical activity, passive smoking, and health literacy. In this study, the prevalence of third-trimester pregnancy depression among pre-pregnancy OWOB women was 34.47%, which is similar to the 30% prevalence reported by Salehi (54) for the second and third trimesters of pregnancy in the same population. Given China’s large population base and the rising proportion of women entering pregnancy with OWOB [according to the 1.13 million live births reported in Guangdong Province in 2024, the number of pre-pregnancy OWOB women suffering from depression in third-trimester pregnancy in Guangdong alone is as high as 98,000 (8)], the mental health of pre-pregnancy OWOB pregnant women should be highly valued by maternal and child health care personnel.

In this study, 93.20% of participants were overweight before pregnancy, while 6.80% were obesity. The findings predominantly reflect the situation of pre-pregnancy overweight women. In this study, educational level was one of the influencing factors of third-trimester pregnancy depression among pre-pregnancy OWOB women, consistent with findings from a retrospective study by Liu et al. on 14,329 pregnant women in southwestern China (55). Previous research has also shown that prenatal depression is more prevalent among women with lower educational (19). Our results indicated that women with an education level of junior high school and below were 2.358 times more likely to experience depression in third-trimester pregnancy compared to those with an undergraduate degree and above (95% *CI*: 1.368 ~ 5.744, *p* = 0.043). This may be explained by the link between lower educational and lower socioeconomic status, which is an important risk factor for depression (56). Furthermore, higher education levels are associated with better understanding of pregnancy- and childbirth-related health knowledge, enabling OWOB women to better access and adhere to health guidance. This may help reduce their anxiety about adverse pregnancy outcomes and, in turn, lower the risk of prenatal depression.

Binary logistic regression results revealed the association between career and third-trimester pregnancy depression among pre-pregnancy OWOB women. Compared with healthcare personnel, the risk of third-trimester pregnancy depression increased by 5.534 times (95% *CI*: 1.456 ~ 21.038, *p* = 0.012), 8.659 times (95% *CI*: 1.969 ~ 38.088, *p* = 0.004), and 7.622 times (95% *CI*: 1.703 ~ 34.110, *p* = 0.008) for pregnant women in enterprises or institutions, unemployed, and other careers, respectively, which was consistent with the results of He in 4,564 pregnant women in Beijing (57). Healthcare personnels may be able to manage emotional changes more effectively due to their strong professional knowledge and depression recognition ability. Among all career categories, unemployed women had the highest risk of depression, which aligns with previous findings (20). This may be attributed to individuals with lower socioeconomic status who often lack adequate social support, experience reduced social interactions that can lead to loneliness and low self-esteem, and face financial stress due to limited income (58). In addition, our study found that family income was also an influencing factor of third-trimester pregnancy depression. Women with income of 4,000–5,999 CNY had a 2.633 times higher risk of depression compared to those with income exceeding 15,000 CNY (95% *CI*: 1.024 ~ 6.773 *p* = 0.045). This result further supports the link between economic conditions and maternal mental health and also supported the finding that unemployed women were more vulnerable to prenatal depression.

We also found that having had a previous pregnancy was a risk factor for depression in third-trimester pregnancy among pre-pregnancy OWOB women, and the risk was 2.080 times higher than that of first-pregnancy women (95% *CI*: 1.199 ~ 3.606 *p* = 0.009). Current evidence on the relationship between the number of pregnancies and prenatal depression remains inconsistent. Studies have suggested that first pregnancy may be a risk factor for third-trimester depression (59), while Ngocho reported no significant association between the number of pregnancies and prenatal depression (60). On the other hand, Qi found that multiparous women were at greater risk for anxiety and depression than primiparous women (61). Therefore, the prediction of the first pregnancy may be affected by the characteristics of pregnant women. In this study, non-first-time pregnant women have a higher risk of depression in third-trimester pregnancy, which may be related to their more family and childcare responsibilities. Additionally, previous adverse pregnancy or birth experiences may negatively influence women’s emotional state during subsequent pregnancies (62). OWOB women are more likely to experience adverse outcomes in previous pregnancy, which may aggravate their depression level in subsequent pregnancy. This suggests that maternal healthcare providers should provide more attention and intervention measures in line with the characteristics of pre-pregnancy OWOB pregnant women according to the specific conditions of women, so as to reduce the risk of depression and ensure maternal and child health.

Although an increasing number of studies focused on how mothers cope with depression at the emotional and psychological levels (63, 64), examining the association between lifestyle behaviors and mental health during pregnancy is particular significance for pre-pregnancy OWOB pregnant women. On one hand, pregnant women are generally more motivated and receptive to adopting healthy behaviors compared to non-pregnant periods (65). On the other hand, OWOB pregnant women are often accompanied by one or more unhealthy lifestyles (66). It is important to note that the relationship between lifestyle and depression may be bidirectional: unhealthy lifestyle behaviors may increase the risk of depression, whereas depression itself may impair the ability to maintain healthy behaviors. Within this context, as a non-drug intervention, lifestyle modification is relatively easy to implement. Investigating its relationship with third-trimester depression can help maternal healthcare providers achieve earlier prevention and intervention among OWOB pregnant women in a more cost-effective, safer, and feasible manner.

This study identified DPs as significant influencing factors of depression in third-trimester pregnancy among pre-pregnancy OWOB women. This may be explained by the fact that dietary habits are key lifestyle factors contributing to excessive weight gain (67, 68), which in turn is closely associated with the risk of depression. Specifically, balanced DP was a protective factor for depression, while highly processed DP increased the risk of depression; correspondingly, we found that the balanced DP was negatively correlated with pre-pregnancy BMI, while the highly processed DP was positively correlated with pre-pregnancy BMI (*p* < 0.05). The balanced DP in our study was rich in calcium, vitamin B complex, and protein, which are known to support nervous system stability, reduce anxiety, and alleviate depression. Both the quality and quantity of dietary protein are important. In this study, red meat was the primary protein source in the balanced DP, supporting prior evidence linking iron status to depression (69). In contrast, the highly processed DP was rich in high-sugar, high-fat, and low-fiber foods, which can easily lead to physiological reactions closely related to depression, such as blood glucose fluctuations, insulin resistance, and chronic inflammation (70). Alcohol intake, also included in this DP, may further exacerbate depression (71). Moreover, women who favor highly processed DP may experience internal conflict—motivated to improve health but simultaneously feeling frustration or guilt over their food choices—which may intensify psychological stress and contribute to depression. With the growing adoption of mobile health technologies, maternal healthcare providers can utilize smartphone applications and digital platforms to monitor the daily diet of OWOB pregnant women (72). In addition, encouraging the involvement of family members, especially partners, in healthy eating has been shown to improve dietary adherence and enhance intervention effectiveness (73). Such strategies may help shift dietary preferences toward more nutritional balanced DP and reduce dependence on highly processed foods in this high-risk population.

Physical activity level was also found to be associated with depression in third-trimester pregnancy. Consistent with previous research, our findings support the inverse association between physical activity and prenatal depression (23). Although moderate physical activity was considered as a promising non-drug therapy to alleviate depression (74), the mean PARS-3 score among participants in this study was 7.11 ± 5.47, indicating an overall low level of physical activity. This may be attributed to the physical limitations of OWOB pregnant women (such as joint burden, poor activity endurance), which can hinder their ability to engage in high-intensity or regular exercise (75). These barriers may confine the direct mental health benefits of physical activity. Therefore, maternal healthcare providers should offer individualized activity recommendations that consider both the physical risks and the specific capacities of OWOB pregnant women (76). For example, OWOB pregnant women are less likely to engage in physical activity during pregnancy than normal-weight women (77). Low-intensity leisure activities, such as walking, may be more feasible and sustainable to increase their activity levels (78). At the same time, well-designed and adequately powered randomized trials are needed to further investigate the effects of various intensities of physical activity on the mental status of OWOB pregnant women.

Sleep disorder was identified as a risk factor for depression among pre-pregnancy OWOB pregnant women. Physiological, psychological, and lifestyle changes during pregnancy may interfere with sleep quality (79). Additionally, OWOB may also lead to obstructive sleep apnea (OSA), affecting sleep quality, and triggering or exacerbating depression through multiple pathways (24). Dominguez have noted that existing OSA screening tools may lack sensitivity in extremely obese pregnant women, and further research is needed to adjust the evaluation criteria and thresholds (80). Wearable sleep-monitoring technologies represent a promising tool for identifying sleep disorder in OWOB pregnant women, offering maternal healthcare providers more objective data to inform diagnosis and management strategies (81). In our study, health literacy was found to be negatively associated with depression, consistent with findings in other populations (82). Pregnant women with low health literacy may perceive depressive symptoms as a normal emotional response and ignore seeking help, whereas those with higher health literacy are usually better able to recognize emotional changes and respond proactively (83). Health literacy is the foundation of health-promoting behaviors. When providing lifestyle intervention guidance for the mental status of pre-pregnancy OWOB pregnant women, maternal healthcare providers should also focus on enhancing their health literacy. One effective approach is the Teach-back method, which involves encouraging pregnant women to repeat key information in their own words. This approach helps confirm understanding and strengthens their ability to follow health recommendations (84).

In our study, passive smoking among pre-pregnancy OWOB women was not statistically associated with third-trimester depression. Although many studies reported second-hand smoke exposure as a risk factor for depression, several well-conducted investigations have not found a significant link (85–87). A large-scale German study involving 28,000 never-smokers reported no significant association between second-hand smoke exposure and depression among women, and even observed an inverse relationship among men (88). Such inconsistent findings may reflect differences in exposure assessment, population or contextual factors, or residual confounding. For instance, individuals exposed to second-hand smoke may be more frequently involved in social interactions, which may be protective for the development of depression (89). These considerations may help explain why no significant association was observed in our sample. However, this does not imply that passive smoking has no effect on third-trimester depression; more precise exposure measurement or higher thresholds of exposure may be needed to identify or elicit such an effect.

This study is associated with certain limitations. First, due to the cross-sectional design, depression and demographic or lifestyle factors may not necessarily indicate a cause-effect relationship. Second, the prevalence of clinically diagnosed prenatal depression in our OWOB pregnancy population was not determined as mental status examinations and biomarker measurements were not performed. Third, the sample was unbalanced between overweight and obesity subgroups; therefore, the findings primarily reflect overweight women and should be generalized to obese women with caution. Finally, potential unmeasured confounders, such as social support, psychiatric history, and obstetric complications, may also have influenced the results and should be addressed in future research.

## 5 Conclusion

This study revealed that about one-third of pre-pregnancy OWOB women experienced depression in third-trimester pregnancy. It is important to pay attention to associated factors of depression in these women. In addition to demographic factors, several modifiable lifestyle behaviors—including DP, physical activity, sleep quality, and health literacy—were found to be associated with depression. These findings not only contribute to identifying high-risk individuals but also provide practical guidance for the development of subsequent interventions. Given the modifiable and cost-effective nature of lifestyle factors, maternal healthcare providers are encouraged to consider them as key entry points for addressing depression among pre-pregnancy OWOB women. By combining mental health management with lifestyle intervention, maternal and child health can be guaranteed in a more economical, safe and accessible way.

## Data Availability

The raw data supporting the conclusions of this article will be made available by the authors, without undue reservation.
