# Supplementary material for: The prevalence and associated factors of third-trimester pregnancy depression in pre-pregnancy overweight and obesity women: a cross-sectional study in Guangdong, China
Source: Front Public Health. 2025 Oct 14;13:1687185. doi: 10.3389/fpubh.2025.1687185 (PMC12558768; doi:10.3389/fpubh.2025.1687185)
Supplement: Supplementary file 1 [file Table_1.docx]

**Table S1** Food items included in each food group

| No. | Foods or food groups | Food items from FFQ |
| --- | --- | --- |
| 1 | Rice and flour-based food | Rice, rice noodles, porridge and other rice products; noodles, steamed bread and other wheat products |
| 2 | Grains and tubers | Corn, millet, oats, and other grain products; Potato, sweet potato, yam, and other tuber products |
| 3 | Dark-colored vegetables | Spinach, lettuce, broccoli, carrot, pumpkin, purple kale, etc. |
| 4 | Light-colored vegetables | Cabbage, white radish, winter melon, cucumber, bean sprouts, etc. |
| 5 | Fruits | Apples, pears, oranges, tangerines, oranges, pomelos, bananas, peaches, plums, grapes, strawberries, pineapples, etc. |
| 6 | Red meat | Beef or veal, lamb, pork, mixed meat dishes, etc. |
| 7 | Poultry | Chicken, duck, goose, pigeon, mixed poultry dishes, etc. |
| 8 | Freshwater seafood | River fish, river prawns, crayfish, field snails, soft-shelled turtle, etc. |
| 9 | Seafood | Fishes, shrimps, crabs, shellfishes, kelp, nori, abalone, jellyfish, squid, sea cucumber, etc. |
| 10 | Edible mushrooms | Shiitake mushrooms, enoki mushroom, wood ear mushroom, silver fungus, etc. |
| 11 | Eggs | Eggs, mixed egg dishes. |
| 12 | Dairy products | Milk, yoghurt, cheese, milk powder, etc. |
| 13 | Beans products | Soy bean, mung bean, red bean, soybean milk, tofu, doupi, etc. |
| 14 | Nuts | Peanuts, melon seeds, walnuts, cashews, etc. |
| 15 | Organ meats | Liver, intestine, tripe, other livestock or poultry offal. |
| 16 | Processed snacks | Biscuits, candy, chocolate, cake, bread, etc. |
| 17 | Alcoholic and carbonated beverages | Alcohol, carbonated beverages, caffeinated beverages |
| 18 | Fried and pickled foods | French fries, deep-fried dough sticks, instant noodles, and other fried products; Pickles, bacon, smoked meat, sausage and other cured smoked products |
